# High-Frequency Irreversible Electroporation Alters Proteomic Profiles and Tropism of Small Tumor-Derived Extracellular Vesicles to Promote Immune Cell Infiltration

**DOI:** 10.3390/cells14221782

**Published:** 2025-11-13

**Authors:** Kelsey R. Murphy, Kenneth N. Aycock, Spencer Marsh, Liping Yang, Jonathan Hinckley, Aubrie Selmek, Robert Gourdie, Shay Bracha, Rafael V. Davalos, John H. Rossmeisl, Nikolaos G. Dervisis

**Affiliations:** 1 Department of Biomedical and Veterinary Sciences, Virginia-Maryland College of Veterinary Medicine, Blacksburg, VA 24061, USA; 2 Department of Biomedical Engineering and Mechanics, Virginia Tech, Blacksburg, VA 24061, USA; kenneth.aycock@intusurg.com (K.N.A.); gourdier@vt.edu (R.G.); davalos@vt.edu (R.V.D.); 3 Fralin Biomedical Research Institute, Virginia Tech Carilion School of Medicine, Virginia Tech, Roanoke, VA 24016, USA; srmarsh@vt.edu; 4 Center for Heart and Reparative Medicine Research, Virginia Tech, Roanoke, VA 24016, USA; 5 Department of Chemistry, Oregon State University, Corvallis, OR 97331, USA; liping.yang@oregonstate.edu; 6 Department of Small Animal Clinical Sciences, Virginia-Maryland College of Veterinary Medicine, Blacksburg, VA 24061, USA; hinckley@vt.edu (J.H.); jrossmei@vt.edu (J.H.R.); ndervisi@purdue.edu (N.G.D.); 7 Department of Veterinary Clinical Sciences, College of Veterinary Medicine, The Ohio State University, Columbus, OH 43210, USA; selmek.5@osu.edu (A.S.); bracha.2@osu.edu (S.B.); 8 Translational Biology Medicine and Health Graduate Program, Virginia Tech, Roanoke, VA 24016, USA; 9 Department of Emergency Medicine, Virginia Tech Carilion School of Medicine, Virginia Tech, Roanoke, VA 24016, USA; 10 ICTAS Center for Engineered Health, Virginia Tech, Kelly Hall, Blacksburg, VA 24061, USA; 11 Department of Biomedical Engineering, Georgia Tech-Emory University, Atlanta, GA 30332, USA; 12 Department of Internal Medicine, Virginia Tech Carilion School of Medicine, Roanoke, VA 24016, USA

**Keywords:** electroporation, cancer, brain tumor, blood–brain barrier, immunomodulation, tumor microenvironment, tumor ablation, glioma, extracellular vesicles, exosomes 10

## Abstract

High-frequency irreversible electroporation (H-FIRE) is a nonthermal tumor ablation technique that disrupts the blood–brain barrier (BBB) in a focal and reversible manner. However, the mechanisms underlying this disruption remain poorly understood, particularly the role of small tumor-derived extracellular vesicles (sTDEVs) released from ablated tumor cells. In this study, we investigate the proteomic and functional alterations of sTDEVs released from F98 glioma and LL/2 Lewis lung carcinoma cells following H-FIRE ablation. Mass spectrometry analysis revealed 108 unique proteins in sTDEVs derived from ablative doses of H-FIRE, which are capable of disrupting the BBB in an in vitro model. Proteomic analysis of TDEVs highlights key changes in pathways related to integrin signaling, Platelet-derived growth factor receptor (PDGFR) signaling, and ubiquitination, which may underline their interactions with brain endothelial cells. These “disruptive” sTDEVs exhibit enhanced tropism for cerebral endothelial cells both in vitro and in vivo, where they persist in the brain longer than sTDEVs released after non-ablative H-FIRE doses. Notably, when introduced into a healthy Fischer rat model, disruptive sTDEVs are associated with increased recruitment of Iba1+ immune cells, suggesting a potential role in modulating post-ablation immune responses. However, despite their altered protein composition, these vesicles do not directly increase BBB permeability in vivo. This study is the first to demonstrate that electroporation-based tumor ablation significantly alters the composition and functionality of tumor-derived extracellular vesicles, potentially influencing the tumor microenvironment post-ablation. These findings have important implications for developing multimodal treatment strategies that combine H-FIRE with systemic therapies to enhance efficacy while managing the peritumoral microenvironment.

## 1. Introduction

Malignant brain tumors carry an annual mortality rate of ~4.5 for every 100,000 people [1], including mortality associated with both primary brain tumors and brain metastases (BM). The most common and deadly primary brain tumor, glioblastoma (GBM), is invariably fatal [2] despite a multimodal therapeutic approach including aggressive surgical resection, temozolomide chemotherapy, and radiation therapy [3]. Invasive malignant cells prevent complete surgical resection, ultimately resulting in tumor recurrence [4]. Additionally, the peritumoral blood–brain barrier (pBBB) shields the infiltrative cancer cell populations from systemic delivery of chemotherapeutics, further contributing to tumor recurrence [5]. In addition to primary brain tumors, BM are the most prevalent brain cancer in adults and present ten times more frequently than primary brain tumors [6]. BM comprise over 50% of all brain cancers [5], commonly arising from primary lung, breast, and renal tumors. BM therapy includes a combination therapy including stereotactic radiosurgery (SRS), surgical resection, whole-brain radiotherapy (WBRT), or chemotherapy [7]. Even still, BM are often considered terminal with a 6-month median survival after treatment [5].

The still-limited survival for BM and GBM patients with current treatment regimens demands the development of selective and minimally invasive treatment options that address current treatment challenges, including protection of malignant cells from systemic therapies by the BBB, cancer cell invasion into healthy brain tissue, and resistance. Energy-based tumor ablation therapeutics carry promise in these regards. High-frequency irreversible electroporation (H-FIRE) is a novel, electric field-based tumor ablation modality that is not dependent on thermal mechanisms. During H-FIRE treatment, needle electrodes are inserted into the tumor, and high-frequency bipolar electric pulses are delivered directly into the target neoplastic tissue. As a result, the transmembrane potential of tumor cells is elevated, irreversible cell membrane pores form, and cell death occurs within a sharply delineated treatment volume [8]. H-FIRE produces uniform ablations without significant thermal effects while minimizing muscle contractions observed during standard irreversible electroporation (IRE) [9]. H-FIRE is clinically efficacious in clinical trials of primary brain tumor-bearing dogs while sparing surrounding healthy brain tissue, suggesting that H-FIRE may represent a minimally invasive treatment option for people with brain cancer [10].

An added therapeutic benefit of H-FIRE tumor ablation is focal and transient disruption of the pBBB in a penumbra around the ablated tumor tissue. This can be exploited to distribute systemic therapeutics to infiltrative tumor cells beyond the ablation borders [10,11,12]. A complete understanding of the mechanisms of H-FIRE-induced pBBB disruption is critical for the design of multimodal therapeutic approaches utilizing H-FIRE and peritumoral delivery of systemic anticancer therapeutics. Tumor-derived extracellular vesicles are a highway of communication between the tumor and its immediate microenvironments, and various anticancer therapies are known to impact tumor-derived extracellular vesicle cargo and function to mediate various changes in the tumor microenvironment after treatment. Zhao et al. have demonstrated that focused ultrasound hyperthermic ablation alters the proteomic cargo of extracellular vesicles to disrupt the BBB [13]. This phenomenon has never been studied after H-FIRE tumor ablation and may represent a mechanism of pBBB disruption after H-FIRE ablation of brain tumors. Using primary and metastatic brain tumor cell models (F98 glioma and LL/2 Lewis lung carcinoma cells, respectively), we have previously shown that small tumor-derived extracellular vesicles (sTDEVs) released after treatment of brain cancer cells with 3000 V/cm H-FIRE increased permeability of the BBB endothelium in vitro [14].

Here, we hypothesize that these post-H-FIRE, in vitro BBB-disruptive sTDEVs include exosomes, a specific subtype of extracellular vesicle. Further, we hypothesize that H-FIRE treatment alters the proteomic profiles of such sTDEVs, resulting in internalization by cerebral endothelial cells both in vitro and in vivo and permeabilization of the in vivo BBB. We treat F98 glioma and LL/2 Lewis lung carcinoma cells in vitro with H-FIRE, isolate sTDEVs, and characterize exosomal signatures via Western blot. To determine whether alterations in exosomal proteomic cargo may drive these functional modifications of sTDEVs observed after tumor cell treatment with H-FIRE, we use label-free, bottom-up mass spectrometry to characterize the proteomic profiles of the sTDEVs released after H-FIRE treatment of glioma cells. To investigate a potential mechanism of the BBB endothelium permeabilization demonstrated previously [14], we fluorescently label sTDEVs released after H-FIRE treatment and use confocal microscopy to examine post-H-FIRE sTDEV internalization by cerebral endothelial cells in vitro. Finally, using a healthy rat model, we investigate whether the sTDEVs released after H-FIRE treatment of glioma cells exhibit brain and cerebral endothelial cell tropism, modulate immune cell infiltration, and increase permeability of the BBB in vivo.

## 2. Materials and Methods

### 2.1. Cell Culture

Rat F98 glioma cell line (ATCC, CRL-2397), murine LL/2 (LLC1) Lewis lung carcinoma cell line (ATCC, CRL-1642), and murine bEnd.3 cerebral endothelial cell line (ATCC, CRL-2299) were grown in DMEM (ATCC, 30-2002) supplemented with 1% penicillin-streptomycin (Thermo Fisher Scientific, Gibco, Waltham, MA, USA) and 10% fetal bovine serum (Thermo Fisher Scientific, Gibco). Cells were grown at 37 °C and 5% CO_2_ and passaged regularly. All cells used were between passages 4 and 12 and detached for passaging using TrypLE Express Enzyme at room temperature (Thermo Fisher Scientific, Gibco).

### 2.2. H-FIRE Treatment of Tumor Cell Lines

LL/2 and F98 cells were grown to 80% confluence. Twenty-four hours prior to H-FIRE treatment, the media was removed and monolayers were rinsed with Hank’s Balanced Salt Solution (Thermo Fisher Scientific, Gibco) 5 times. DMEM supplemented with 10% exosome-depleted fetal bovine serum (Thermo Fisher Scientific, Gibco) and 1% penicillin-streptomycin (Thermo Fisher Scientific, Gibco) was added to the cells. For H-FIRE treatment, LL/2 and F98 cells were detached with TrypLE, washed, and resuspended in a 5.5:1 ratio of low-conductivity sucrose solution (85 g sucrose, 3 g glucose, 7.25 mL DMEM, and 992.75 mL DI water) to unsupplemented DMEM to a concentration of 4.0 × 10^6^ cells/mL. An amount of 800 mL of cell suspension was added to sterile electroporation cuvettes (4 mm gap) (USA Scientific, Ocala, FL, USA). A custom-built H-FIRE pulse generator delivered H-FIRE pulses (VoltMed Inc., Blacksburg, VA, USA). Treatments comprised 200 bursts of square bipolar pulses delivered at a frequency of 1 burst per second. One burst of bipolar pulses consisted of a 2 μs positive pulse, a 5 μs interphase delay, a 2 μs negative pulse, and a 5 μs interpulse delay (2-5-2 μs pattern), which was repeated for a total of 100 μs energized time per burst. An oscilloscope was used to monitor current and treatment voltage. H-FIRE treatments were delivered at electric field strengths of 0, 1500, and 3000 V/cm. These electric fields were selected based on our previous work demonstrating sub-ablative (1500 V/cm) and ablative (3000 V/cm) effects on these cell lines [15]. Following H-FIRE treatment, cell suspensions were incubated at a temperature of 37 °C for 20 min. Suspensions were then centrifuged at 300× *g* for 6 min, and supernatants were stored at −80 °C for later use. H-FIRE treatments of tumor cells for all experiments below were performed on the same day for consistency between experiments.

### 2.3. Isolation of Tumor-Derived Extracellular Vesicles

Post-H-FIRE supernatants were thawed. Pre-wetted 0.22 μm polyester syringe filters were used to filter the thawed supernatants. Eluates were collected and ultracentrifuged at 100,000× *g* for 1 h at 4 °C (Beckman Optima TLX Ultracentrifuge, TLA 120.2 rotor) (Beckman Coulter, Brea, CA, USA). Following discarding of the supernatant, sTDEV pellets were resuspended in the appropriate buffer for immediate use.

### 2.4. Western Blot

LL/2 and F98 sTDEV pellets were suspended in RIPA buffer with protease inhibitor and incubated on ice for 5 min. Suspensions were centrifuged at 10,000× *g* for 10 min at 4 °C. Supernatants were collected and quantified via Pierce^TM^ BCA Assay (Thermo Fisher Scientific). Quantified lysates (50 μm) were combined with 1X LDS Sample Buffer, 1X Reducing Agent, and 35 µL of ultrapure water. A reducing agent was added to the samples. Samples were then heated at 95 °C for 5 min. Samples (35 µL) and a pre-stained protein ladder were loaded into precast 10% Bis-Tris Mini Protein Gels (NuPAGE) (Thermo Fisher Scientific, Waltham, MA, USA). Gels were electrophoresed with the preprogrammed method for Mini Gels (subselection: NuPAGE Bis-Tris (MOPS)) on a PowerEase Touch Power Supply (Invitrogen, Carlsbad, CA, USA). Proteins were transferred to PDVF membranes in methanol for 30 s. Successful transfer was verified with Pnceau S staining for 5 min. Membranes were blocked with EveryBlot Blocking Buffer (BioRad, Hercules, CA, USA) for 5 min. The membrane was cut into sections and incubated in blocking buffer containing one of the following primary antibodies at 4 °C overnight: anti-GAPDH (Invitrogen, Cat #PA585074, 1:7500), anti-HSP70 (Invitrogen, Cat #MA3006, 1:1000), or anti-moesin (Invitrogen, Cat #PA5-70605, 1.0 µg/mL). Membranes were washed with 1X PBS Tween-20 for 15 min and 3X for 5 min. Membranes were incubated in blocking buffer containing either goat-anti-mouse (Invitrogen, Cat #31430, 1:7500) or goat-anti-rabbit (Invitrogen, Cat #31460, 1:7500) secondary antibodies for 60 min at room temperature. Membranes were developed using a 1:3 mixture of Supersignal West Pico PLUS Chemiluminescent Substrate and Supersignal Femto Maximum Sensitivity Substrate (Thermo Scientific). Membranes were imaged using a ChemiDoc MP Imaging System (BioRad), and both a colorimetric and chemiluminescent image were obtained using the Rapid Auto-Exposure setting.

### 2.5. LC-MS/MS

Post-H-FIRE F98 sTDEV pellets were resuspended in PBS and quantified via BCA assay as previously described. Quantified sTDEVs were pooled to 23 μm sTDEV suspensions at 750 μm/mL. Triplicate sTDEV suspensions per H-FIRE treatment group were sent to the Oregon State University Mass Spectrometry Center for label-free, bottom-up analysis. Proteins were digested by mass spectrometry-grade modified Trypsin (Trypsin Gold, Promega Corporation, Madison, WI, USA) and desalted. Peptides were analyzed using an Orbitrap Fusion Lumos mass spectrometer with a Nano ESI source (Thermo Scientific) coupled with a Waters nanoAcquity UPLC system (Waters, Milford, MA, USA). Mobile phases consisted of LC-MS-grade water containing 0.1% formic acid (solvent A) and 0.1% formic acid in LC-MS-grade acetonitrile (solvent B). Desalted proteolytic products were loaded on a nanoAcquity UPLC 2 G Trap Column (180 μm × 20 mm, 5 μm) for 5 min with 3% solvent B at a flow rate of 5 μL/min. A nanoAcquity UPLC RPeptide BEH C18 column (100 μm × 100 mm, 1.7 μm) was employed for peptide separation. Peptide elution was executed using a 120-minute gradient in which solvent B was increased from 3 to 10% at 3 min, reached 30% at 105 min, 90% at 108 min, held at 90% for 4 min, decreased to 3% at 113 min, and held at 3% until 120 min. The LC flow rate was 500 nL/min. All spectral data were acquired in the positive ion mode with a spray voltage of 2400 V and an ion transfer tube temperature of 300 °C. MS spectra were acquired by the Orbitrap analyzer with 120 K resolution at *m*/*z* 200. The ion trap analyzer acquired all MS/MS spectra in collision-induced dissociation fragmentation mode. Automatic gain control target was set to 4.0 × 10^5^ and 10^4^ for precursor ions and product ions, respectively. Mass tolerances were set at ±10 ppm for precursor ions and 0.6 Da for product ions. Thermo Scientific Proteome Discoverer 2.2 software was used for raw data analysis and for searching against the Uniprot *Rattus norvegicus* protein database using the Sequest HT search engine. Carbamidomethylation of cysteine and oxidation of methionine were specified as static modification and dynamic modification, respectively, with up to two missed cleavages allowed. Protein identifications were filtered using a target-decoy strategy to achieve an overall false discovery rate (FDR) < 1% at the protein level. Protein quantification was performed by label-free MS1 intensity-based quantification; spectral counting was not used. Missing intensity values, assumed to represent signals below the detection limit, were imputed globally per protein as one-fifth of the minimum nonzero intensity observed across all samples. This left-censoring approach minimizes bias in downstream log transformation and differential testing. Processed data were filtered by interquartile range, normalized by sample sum, and log-transformed before statistical analysis in MetaboAnalyst 5.0. “Non-disruptive” labels were assigned to 0 V/cm and 1500 V/cm samples, and “disruptive” labels were assigned to 3000 V/cm samples. Differentially abundant proteins were identified by unpaired two-tailed t-tests with Benjamini–Hochberg correction (FDR < 0.05). Partial least squares-discriminant analysis (PLS-DA) was performed in MetaboAnalyst 5.0 to visualize global variance among treatment groups. Proteins were considered absent from sTDEVs within a specific H-FIRE dose if all three replicates for that dose were below the LoD threshold. Proteins unique to or shared between H-FIRE treatment groups were identified and converted to gene symbols for Enrichr Pathway Analysis (NCI Nature 2016, Bioplanet 2019, Elsevier Pathway Collection, and Panther 2016 libraries). The raw MS data were uploaded to Purdue University Research Repository (PURR) (DOI: 10.4231/J1N3-NW94).

### 2.6. Fluorescent Labeling of Tumor-Derived sTDEVs

Post-H-FIRE supernatants were filtered through 0.22 μm polyester syringe filters (pre-wetted). CFSE in anhydrous DMSO was added to filtration eluates to a final concentration of 8 mM and incubated in the dark at 37 °C. Suspensions were placed on ice to stop labeling and ultracentrifuged for 1 h at 100,000× *g* at 4 °C (Beckman Optima TLX Ultracentrifuge, TLA 120.2 rotor). Supernatants were discarded, PBS was added to sTDEV pellets, and sTDEV pellets were left overnight at 4 °C in the dark to loosen the pellet. LL/2 and F98 sTDEVs were triturated to resuspend in PBS and washed at 100,000× *g* for 1 h at 4 °C. Supernatants were discarded, and CFSE-labeled sTDEV pellets were resuspended in filtered PBS for confirmation of CFSE labeling via flow cytometry (Cytoflex, Beckman Coulter, Brea, CA, USA), in DMEM supplemented with 10% fetal bovine serum and 1% penicillin-streptomycin for in vitro uptake experiments, or in 0.9% sterile saline for in vivo experiments.

### 2.7. Confocal Microscopy of Cerebral Endothelial Cell Uptake of CFSE-Labeled sTDEVs

Bend.3 cerebral endothelial cells were grown to 90% confluence in 4-well Lab-Tek^TM^ chamber slides (Thermo Scientific) in DMEM supplemented with 1% penicillin-streptomycin (Thermo Fisher Scientific, Gibco) and 10% fetal bovine serum (Thermo Fisher Scientific, Gibco). Cells were incubated at 37 °C in a humidified environment containing 5% CO_2_. The media was changed every two days. Media was aspirated from bEnd.3 cells, and monolayers were rinsed with Hank’s Balanced Salt Solution (Thermo Fisher Scientific, Gibco). Wells of bEnd.3 monolayers were exposed to 10 μg of CFSE-labeled LL/2 or F98 sTDEVs suspended in complete DMEM in triplicate. Monolayers were incubated for 24 h in the dark at 37 °C and 5% CO_2_. At indicated timepoints, media were aspirated, and monolayers were gently rinsed three times with Hank’s Balanced Salt Solution (Thermo Fisher Scientific, Gibco). Monolayers were incubated protected from light with 5 μm/mL Hoechst 33258 (Thermo Fisher Scientific) in PBS for 10 min at room temperature. The stain was removed, and the monolayers were rinsed three times with Hank’s Balanced Salt Solution. Monolayers were fixed in 4% paraformaldehyde for 10 min at room temperature. Paraformaldehyde was removed, monolayers were rinsed with PBS, and slides were coverslipped with Fluoromount-G mounting medium (Thermo Fisher Scientific). Slides were stored for a maximum of 2 h in the dark at 4 °C prior to confocal microscopy. Cover-slipped chamber slides were imaged using a Zeiss LSM 700 Confocal Microscope (Carl Zeiss Microscopy, Jena, Thuringia, Germany) with ZEN software (Version 3.11) using 405 and 455 lasers. Images were captured using the 40× objective at the same exposure to compare changes in CFSE (sTDEV):Hoechst intensity ratios. A minimum of 5 images was quantified per bEnd.3 well. For each image, mean intensity values were quantified for each channel, and CFSE:Hoechst intensity ratios were calculated.

### 2.8. In Vivo Delivery of CFSE-Labeled Post-H-FIRE Tumor-Derived sTDEVs

This study was performed in accordance with the principles of the Guide for the Care and Use of Laboratory Animals and was approved by the Institutional Animal Care and Use Committee (IACUC #22-225). Twenty 8–9-week-old male Fischer rats, weighing between 170 and 215 g, were used. Prior to surgery, rats received a subcutaneous 0.65 mg/kg injection of buprenorphine (Ethiqa XR, Fidelis Pharmaceuticals LLC, North Brunswick, NJ, USA). Rats were anesthetized using isoflurane induction (3–4%:95% isoflurane:oxygen mixture). Animals were maintained with isoflurane (2–3.5%:95% isoflurane:oxygen) via nosecone. The dorsum of the head, extending from the intercanthal area to the cranial cervical region, was clipped and prepared for aseptic surgery with alcohol and chlorohexidine scrubs. Anesthetized rats were transferred to a small animal stereotactic head frame (Model 1350M, David Kopf Instruments, Tujunga, CA, USA). A unilateral rostrotentorial surgical approach was performed on the skull. A high-speed electric drill (Dremel 3000 Series, Mount Prospect, IL, USA) with a 2.4 mm diameter round burr was used to create a unilateral parietal craniectomy defect on the left aspect of the skull of each rodent. A 33-gauge Hamilton 1700 series gas-tight syringe (Hamilton Laboratory Products, Reno, NV, USA) loaded with 100 µg of CFSE-labeled F98 sTDEVs in 100 µL sterile saline, or saline vehicle control, was loaded into a microfusion syringe pump (Harvard Apparatus Pump 11 Elite, Grayline Medical Supplies, Houston, TX, USA) attached to the stereotactic headframe. The syringe was advanced into the left striatum using stereotactic coordinates referenced to the location of the needle to a 5 mm exposure. The microfusion pump was set to deliver the 100 µL intracranial injection at 5 µL/min. After the conclusion of the infusion, the syringe was removed from the brain, and the surgical site was closed using 4-0 monocryl uninterrupted skin sutures (Ethicon). Rats were recovered from anesthesia and monitored until their predetermined survival endpoints of 4 h or 24 h post-infusion. Treatment groups designated for MRI were as follows: saline vehicle 4 h (n = 3), 0 V/cm sTDEVs 4 h (n = 3), 0 V/cm sTDEVs 24 h (n = 3), 3000 V/cm sTDEVs 4 h (n = 3), and 3000 V/cm sTDEVs 24 h (n = 3). An additional n = 5 were designated for Iba1+ confocal microscopy investigation, with n = 3 receiving 0 V/cm sTDEVs and n = 2 receiving 3000 V/cm sTDEVs and sacrificed at the 4 h timepoint.

### 2.9. Magnetic Resonance Imaging

One hour prior to the survival endpoints, animals designated for MRI (n = 24) were anesthetized and received an intraperitoneal injection of a solution of Evans Blue Dye (EBD) (75 mg/kg of 2.5% Evans Blue Dye) and gadoteridol (Gd) (0.1 mmol/kg) (ProHance^®^, Bracco, Milan, Lombardy, Italy) to assess BBB permeability. Rats were recovered from anesthesia, and one hour later (4 h and 24 h post-infusion), rats were re-anesthetized, and brain MRI images were obtained in a 9.4 Tesla Biospec 94/20 USR high-field MRI scanner (Bruker Ettlingen, Baden-Wurttemberg, Germany) with an 86 mm volume coil and 2 mm surface coil. T1-weighted images were obtained by FLASH sequence using the following parameters: TE = 3 ms, TR = 210 ms, FA = 70°, slice number = 16, slice thickness = 0.8 mm, matrix = 256 × 256, FOV = 25.6 mm × 25.6 mm, bandwidth = 39,682.5 Hz, average = 3, and scan time = 2 min. T1-weighted 3D scans were obtained by 3D FLASH sequence using the following parameters: TE = 3 ms, TR = 50 ms, FA = 20°, matrix = 256 × 256 × 16, FOV = 25.6 mm × 25.6 mm × 12.8 mm, bandwidth = 27,777.8 Hz, average = 1, and scan time = 3 min. T2-weighted spin echo was obtained by RARE sequence using the following parameters: TE = 33 ms, TR = 2500 ms, slice number = 16, slice thickness = 0.8 mm, echo spacing = 11 ms, RARE factor = 8, matrix = 256 × 256, FOV = 25.6 mm × 25.6 mm, bandwidth = 33,333.3 Hz, average = 6, and scan time = 8 min. Contrast-enhancing lesion volumes (CEV), representative of BBB disruption, were measured in each rat by manually drawing an ROI on contiguous transverse GdT1W MRI images that contained regions that were hyperintense on GdT1W MRI in the treated hemisphere relative to the contralateral side. The subsequent CEV was determined with the volumetric calculator tool in commercial image analysis software (Osirix MD v14.1.1, Pixmeo SARL, Geneva, Switzerland).

### 2.10. Tissue Processing

Anesthetized animals that were designated for MRI (n = 24) were humanely euthanized immediately following MRI via intraperitoneal pentobarbital (780 mg) overdose (Fatal Plus, Vortech Pharmaceuticals, Dearborn, MI, USA). Anesthetized animals that did not receive Evans Blue dye and did not undergo MRI (n = 5) were humanely euthanized 24 h following surgery via intraperitoneal pentobarbital (780 mg) overdose (Fatal Plus, Vortech Pharmaceuticals). Brains, livers, and spleens were harvested and rinsed in cold PBS. Brains were placed in an adult rodent matrix slicer (Ted Pella Inc. Redding, CA, USA) and serially sectioned in the transverse plane in 5 mm intervals. Each transverse slice was embedded in O.C.T. medium and stored at −80 °C for cryopreservation. Tissue blocks in the plane of the injection site were identified based on gross visualization of blood or Evans Blue Dye on the dorsal surface of the brain. Tissue blocks of interest were serially sectioned (20 mm thickness) at −20 °C (Leica CM1850, Leica Biosystems, Wetzlar, Hesse, Germany), mounted onto positively charged microscope slides, and stored at −80 °C until microscopy.

### 2.11. Confocal Microscopy

Tissue-mounted slides were thawed to room temperature and fixed for 10 min at room temperature in 4% paraformaldehyde. Slides were washed 3 times for 5 min in wash buffer (PBS with 0.5% Tween) (Thermo Fisher Scientific). Slides were blocked for one hour at room temperature in 3% donkey serum (Abcam) and incubated overnight at 4 °C with either goat anti-rat polyclonal anti-CD31/PECAM-1 antibody (AF3628, 1:200) (Novus Biologicals, Centennial, CO, USA) or goat anti-rat polyclonal anti-AIF1/Iba1 antibody (NB100-1028, 1:200) (Novus Biologicals). Slides were washed 3 times for 5 min in wash buffer and incubated for 1 h at room temperature with donkey anti-goat IgG AlexaFluor^TM^ 555-conjugated secondary antibody (A-21432, 1:300) (Thermo Fisher Scientific). Slides were washed 3 times for 5 min in wash buffer and stained with 5 μm/mL Hoechst solution for 10 min at room temperature. Slides were washed 3 times for 5 min in wash buffer and rinsed briefly in PBS. Slides were coverslipped using Fluoromount-G mounting medium (Thermo Fisher Scientific) and sealed. Slides were stored for a maximum of 2 h in the dark at 4 °C prior to confocal microscopy. Slides were imaged using a Zeiss LSM 700 Confocal Microscope with ZEN software using 405, 455, 555, and 647 lasers for detection of Hoechst, sTDEVs, CD31, and Evans Blue Dye, respectively. The region of interest was identified in the left hemisphere 5 mm from the dorsal surface of the brain. Images were captured using the 100× objective at the same exposure to compare changes in CFSE (sTDEV):Hoechst intensity ratios, or qualitative spatial visualization of sTDEVs relative to Evans Blue Dye and CD31+ cells. Images were captured using the 40× objective at the same exposure to compare changes in Iba1+ cells compared to CFSE. Laser power was set to 2.0, the AU pinhole size was 1.0, and the gain was set to 650. Image acquisition parameters were held constant across all groups, and analyses were performed to enable relative comparisons of fluorescence intensity rather than subcellular localization. For the quantification of the sTDEV signal, a minimum of 10 images was quantified per tissue section. For each image, mean intensity values were quantified for each channel using ImageJ software (Fiji, version 1.0), and CFSE:Hoechst intensity ratios were calculated.

## 3. Results

### 3.1. Tumor-Derived Extracellular Vesicles Released After H-FIRE Have Exosomal Characteristics

**Figure 1:**
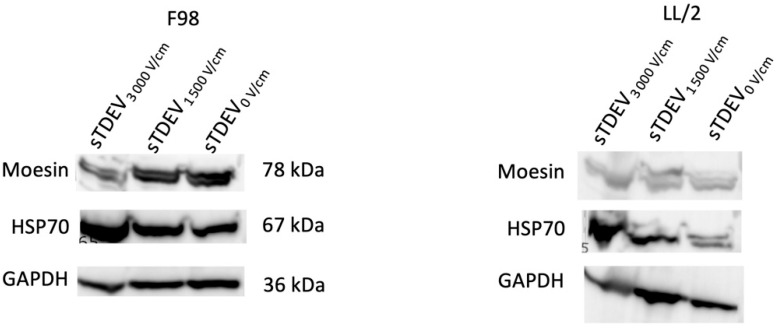
H-FIRE induces the release of tumor-derived extracellular vesicles with exosomal characteristics. F98 and LL/2 Lewis lung carcinoma cells were treated with H-FIRE doses of 0, 1500, and 3000 V/cm. Immediately following treatment, small tumor-derived extracellular vesicles were isolated via filtration and ultracentrifugation. Western blot of small-tumor-derived extracellular vesicles for exosomal markers moesin and HSP70 is shown for F98 (**left**) and LL/2 Lewis lung carcinoma cells (**right**). GAPDH was used as a loading control.

We have previously demonstrated that small tumor-derived extracellular vesicles released after treatment of tumor cells with an ablative 3000 V/cm dose of H-FIRE disrupt the BBB endothelium in vitro [14]. These small tumor-derived extracellular vesicles (sTDEVs) are under 220 nm in size and exhibit typical exosomal morphology by transmission electron microscopy. To determine whether these sTDEV populations include exosomes, we characterized sTDEV populations released by LL/2 Lewis lung carcinoma and F98 glioma cells after treatment with different doses of H-FIRE for exosomal markers (HSP70 and moesin) via Western blot (Figure 1). sTDEVs released after 0 V/cm, 1500, and 3000 V/cm H-FIRE treatment shows expression of exosomal markers HSP70 and moesin, suggesting that the populations of sTDEVs released after H-FIRE treatment include extracellular vesicles consistent with an exosomal phenotype.

### 3.2. H-FIRE Treatment of Glioma Cells Alters the Proteomic Profiles of sTDEVs

**Figure 2:**
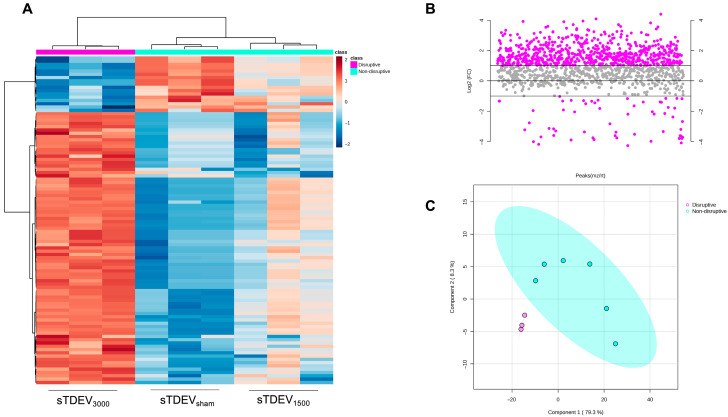
H-FIRE alters the global proteomic profile of sTDEVs. STDEVs were isolated from immediately post-treatment supernatants of F98 glioma cells treated with 0, 1500, and 3000 V/cm H-FIRE and characterized via label-free, bottom-up LC-MS/MS proteomics. “Non-disruptive” grouping includes tumor-derived sTDEVs released after 0 and 1500 V/cm H-FIRE treatment, which previously did not disrupt a Transwell^®^ BBB endothelium model, while “disruptive” grouping includes tumor-derived sTDEVs released after 3000 V/cm H-FIRE treatment, which increased permeability of the Transwell^®^ model. (**A**) Heatmap depicting relative expression of the top 100 proteins with the most significant *p*-values between sTDEVs released following different H-FIRE doses. Statistical results based on the t-test between disruptive and non-disruptive groups. (**B**) Fold change analysis of disruptive/non-disruptive exosomal proteomic payloads with a fold change threshold of 2.0. (**C**) Partial least squares discriminant analysis (PLS-DA) of sTDEVs released after different H-FIRE doses.

We previously demonstrated that sTDEVs released by 3000 V/cm H-FIRE-treated tumor cells increased permeability of the BBB endothelium in vitro, despite the fact that our prior work demonstrates relatively less sTDEVs released after this dose. This suggests that H-FIRE alters the cargo of sTDEVs, rather than increasing sTDEVs abundance, to functionally affect the BBB endothelium. Because sTDEVs released from 3000 V/cm-treated glioma cells significantly increased BBB permeability in vitro, we hypothesized that sTDEVs released by H-FIRE-treated glioma cells has distinct proteomic payloads from sTDEVs released by 0 and 1500 V/cm-treated glioma cells. We performed bottom-up, label-free mass spectrometry proteomics of sTDEVs released by 0, 1500, and 3000 V/cm-treated F98 glioma cells. sTDEVs were grouped according to their effects on the permeability of the BBB endothelium in vitro: sTDEVs released after 0 and 1500 V/cm treatment of glioma cells were labeled “non-disruptive,” while sTDEVs released after 3000 V/cm treatment of glioma cells were labeled “disruptive.” T-tests between disruptive and non-disruptive groups showed that 333 proteins were significantly different between the groups, suggesting that the sTDEVs released after glioma cells are treated with 3000 V/cm H-FIRE, those that increased permeability of the BBB endothelium in vitro, are proteomically distinct. Figure 2A depicts the top 100 proteins with the most significant *p* values. Fold change analysis determined that 58 proteins are significantly decreased and 672 proteins are significantly increased in the “disruptive” sTDEVs compared to the “non-disruptive” sTDEVs (FC ≥ 2.0) (Figure 2B). Partial least squares discriminant analysis (PLS-DA) demonstrates distinct clustering of “disruptive” and “non-disruptive” groups of sTDEVs based on proteomic cargo (Figure 2C).

**Figure 3:**
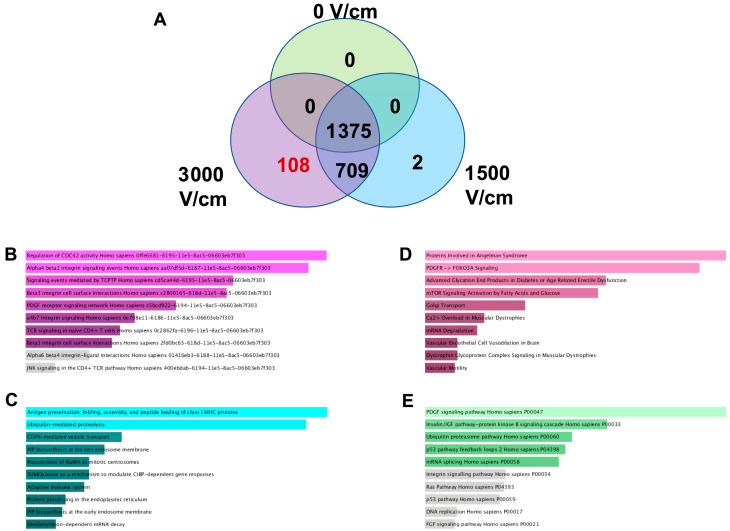
sTDEVs released after ablative H-FIRE doses contain unique proteomic signatures. STDEV released after glioma cell treatment with different doses of H-FIRE was characterized via LC-MS/MS proteomics. (**A**) Venn diagram depicting the number of shared and unique proteins in sTDEVs released after different H-FIRE doses. The 108 proteins unique to sTDEVs released after the 3000 V/cm H-FIRE dose (“disruptive” sTDEVs) were submitted to Enrichr for pathway analysis [16,17,18] with the NCI Nature 2016 library (**B**), Bioplanet 2019 library (**C**), Elsevier Pathway Collection (**D**), and Panther 2016 library (**E**).

From the LC-MS/MS analysis, 1375 proteins were present in sTDEVs released after all three doses of H-FIRE (Figure 3A). Zero proteins were present exclusively in sTDEVs released after 0 V/cm H-FIRE treatment, and zero proteins were common between sTDEVs released after 0 and 3000 V/cm and 0 and 1500 V/cm (Figure 3A). There were two proteins present exclusively in sTDEVs released after 1500 V/cm treatment and 709 proteins common between sTDEVs released after 1500 and 3000 V/cm H-FIRE treatment (Figure 3A). There were 108 proteins that were present exclusively in sTDEVs released after 3000 V/cm H-FIRE treatment (“disruptive” sTDEVs) (Figure 3A, Supplemental Figure S1). In order to determine which proteins make the “disruptive” sTDEVs unique, toward identifying potential mechanisms of the observed sTDEV-mediated functional alterations of the BBB, the 108 proteins exclusively present in the “disruptive” sTDEVs were submitted to Enrichr for pathway analysis [16,17,18]. Pathway analysis was performed in Enrichr using the NCI Nature 2016, Bioplanet 2019, Elsevier Pathway Collection, and Panther 2016 Libraries. The pathways most related to the 108 proteins submitted, as identified by each library, are compiled in Table 1.

### 3.3. Tumor-Derived sTDEVs Released After Ablative Doses of H-FIRE Are Internalized by Cerebral Endothelial Cells In Vitro

**Figure 4:**
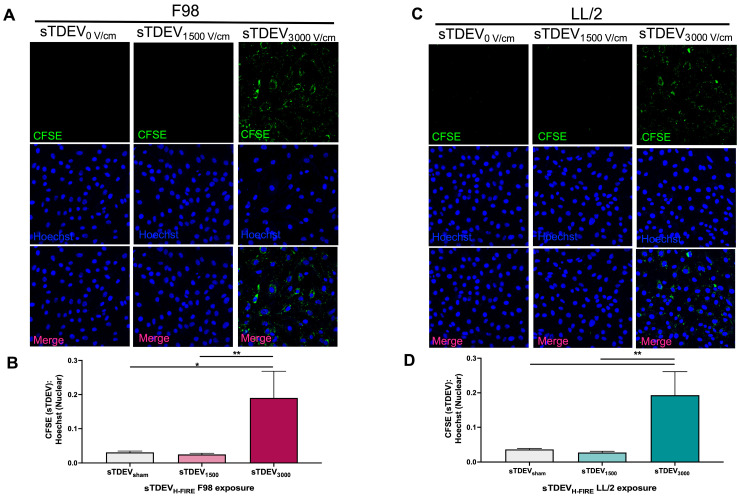
sTDEVs released after ablative H-FIRE doses are internalized by cerebral endothelial cells in vitro. sTDEVs were isolated from supernatants of H-FIRE-treated F98 glioma (**A**,**B**) and LL/2 Lewis lung carcinoma (**C**,**D**) cell lines, fluorescently labeled, and exposed to cerebral endothelial cell monolayers. (**A**) Representative confocal microscopy images of bEnd.3 cerebral endothelial cell monolayers exposed to CFSE-labeled sTDEVs released after 0, 1500, and 3000 V/cm treatment of F98 glioma cells (40× magnification). (**B**) Quantification of post-H-FIRE F98-derived sTDEV uptake by bEnd.3 cerebral endothelial cells. (**C**) Representative confocal microscopy images of bEnd.3 cerebral endothelial cell monolayers exposed to CFSE-labeled sTDEVs released after 0, 1500, and 3000 V/cm treatment of LL/2 Lewis lung carcinoma cells (40× magnification). (**D**) Quantification of post-H-FIRE LL/2-derived sTDEV uptake by bEnd.3 cerebral endothelial cells. Data are presented as mean ± SD. Significant differences are noted between exposure to different sTDEV doses. One-way ANOVA with Tukey’s post hoc test was used for statistical analysis. * *p* ≤ 0.05. ** *p* ≤ 0.01. n ≥ 3 for all groups.

Because sTDEVs released by 3000 V/cm H-FIRE-treated F98 and LL/2 tumor cell lines increased permeability of the BBB endothelium in vitro, and sTDEVs released by 3000 V/cm H-FIRE-treated F98 cells had significantly altered proteomic payloads, we hypothesized that sTDEVs released by H-FIRE-treated tumor cells is internalized by cerebral endothelial cells, allowing them to increase in vitro BBB endothelium permeability. To investigate this potential mechanism of the post-H-FIRE sTDEV-mediated BBB endothelium permeabilization demonstrated previously, we fluorescently labeled sTDEVs released after H-FIRE treatment of F98 and LL/2 tumor cell lines and used confocal microscopy to visualize post-H-FIRE sTDEV internalization by cerebral endothelial cells in vitro (Figure 4). Non-disruptive sTDEVs, released by 0 V/cm-treated (0 V/cm) and sub-ablated (1500 V/cm-treated) F98 glioma cells, were not internalized by cerebral endothelial cells after 24 h of exposure (Figure 4A). STDEVs released by ablated (3000 V/cm-treated) glioma cells, which disrupted the BBB endothelium in vitro, was significantly more internalized by cerebral endothelial cells than sTDEVs released after 0 and 1500 V/cm treatment (*p* < 0.05, *p* < 0.01, respectively) (Figure 4A,B). STDEVs released by 0 V/cm-treated and 1500 V/cm-treated LL/2 Lewis lung carcinoma cells were not internalized by cerebral endothelial cells, while sTDEVs released from 3000 V/cm-treated LL/2 Lewis lung carcinoma cells were significantly more internalized than those released after 0 and 1500 V/cm treatment (*p* < 0.01) (Figure 4C,D).

### 3.4. H-FIRE Treatment of Glioma Cells Alters Brain- and Endothelial Cell Tropism of sTDEVs In Vivo

**Figure 5:**
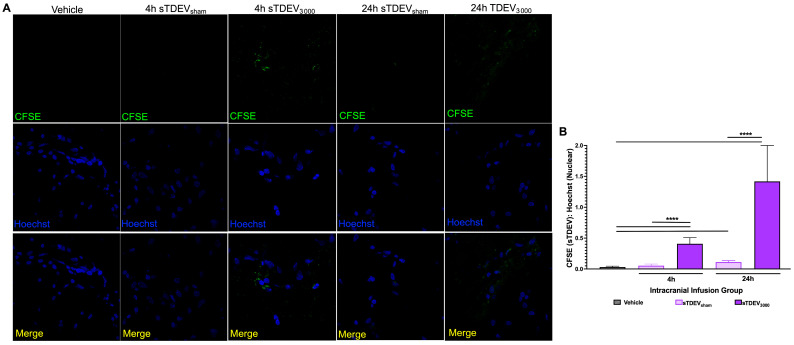
sTDEVs released following ablative H-FIRE doses remain in the brain. sTDEVs were isolated from immediately post-H-FIRE supernatants of 0 V/cm- or 3000 V/cm-treated F98 glioma cells. sTDEVs were labeled with CFSE and administered via intracranial infusion to healthy Fischer rats. Endpoints were 4 and 24 h after infusion. (**A**) Representative confocal microscopy images of brain tissue sections of animals receiving saline vehicle, sTDEVs released after 0 V/cm treatment, and sTDEVs released after 3000 V/cm treatment. sTDEVs are visible in green (CFSE), and nuclei are visible in blue (Hoechst) (100× magnification). (**B**) Quantification of post-H-FIRE F98-derived sTDEV retention in brain tissue. Statistical analysis using one-way ANOVA with Tukey’s post hoc test. **** *p* ≤ 0.0001.

Because the proteomic profiles of disruptive sTDEVs, released from 3000 V/cm H-FIRE-treated glioma cells, were distinct from those of non-disruptive sTDEVs, and the disruptive sTDEVs exhibited increased endothelial cell uptake in vitro, we sought to investigate functional changes in their interactions with the in vivo brain microenvironment. Based on our in vitro results demonstrating increased uptake of disruptive sTDEVs by cerebral endothelial cell monolayers, we hypothesized that disruptive sTDEVs, released from 3000 V/cm H-FIRE-treated glioma cells, may exhibit overall increased accumulation in the brain compared to non-disruptive sTDEVs, which did not show affinity for cerebral endothelial cells in vitro. We treated F98 glioma cells in vitro with 0 and 3000 V/cm doses of H-FIRE and isolated and fluorescently labeled sTDEVs, as was performed previously. sTDEVs were then administered via intracranial infusion to healthy Fischer rat brains in order to recapitulate sTDEV release from an H-FIRE-treated brain tumor. Saline was used as a vehicle control. At 4 and 24 h post-infusion endpoints, tissue sections in the transverse plane of the injection tract were harvested to visualize sTDEVs via confocal microscopy. We obtained confocal images to qualitatively compare tissue-level fluorescence patterns and provide supportive evidence that H-FIRE-treated sTDEVs exhibit altered retention and interaction within the normal brain microenvironment. Both 4 and 24 h after infusion, sTDEVs released by 0 V/cm-treated glioma cells were scarcely visible in the brain (Figure 5A). Visualization of sTDEVs released by 0 V/cm-treated glioma cells in the liver and spleen at both the 4- and 24 h timepoints (Supplemental Figure S2) confirms successful delivery of sTDEVs and suggests that these sTDEVs primarily extravasate and leave the brain. At both 4 and 24 h post-infusion, sTDEVs released by 3000 V/cm H-FIRE-treated glioma cells, the “disruptive” sTDEVs, were clearly visible in the brain (Figure 5A). There was no significant difference in the CFSE:Hoechst signal intensity ratio in the brain between animals receiving sTDEVs released by 0 V/cm-treated glioma cells and animals receiving saline vehicle control at the 4 h timepoint (Figure 5B). This suggests that non-disruptive sTDEVs released by 0 V/cm-treated glioma cells does not significantly accumulate in the brain. Compared to saline vehicle control, the CFSE:Hoechst signal intensity ratio was significantly higher in animals receiving non-disruptive sTDEVs released by 0 V/cm-treated glioma cells at the 24 h timepoint (*p* ≤ 0.0001) (Figure 5B). This is likely due to the breakdown of the fluorescently labeled sTDEVs by 24 h, resulting in the release and spread of CFSE dye into the tissue. Animals receiving infusions of disruptive sTDEVs, released by 3000 V/cm H-FIRE-treated glioma cells, had significantly increased CFSE:Hoechst signal intensity ratios in the brain compared to animals receiving saline vehicle injection and non-disruptive sTDEVs released by 0 V/cm-treated glioma cells at both timepoints (*p* ≤ 0.0001) (Figure 5B). This suggests that H-FIRE alters sTDEVs such that disruptive sTDEVs released by 3000 V/cm H-FIRE-treated glioma cells is retained in the brain tissue significantly more compared to sTDEVs released by 0 V/cm-treated glioma cells (Figure 5B).

**Figure 6:**
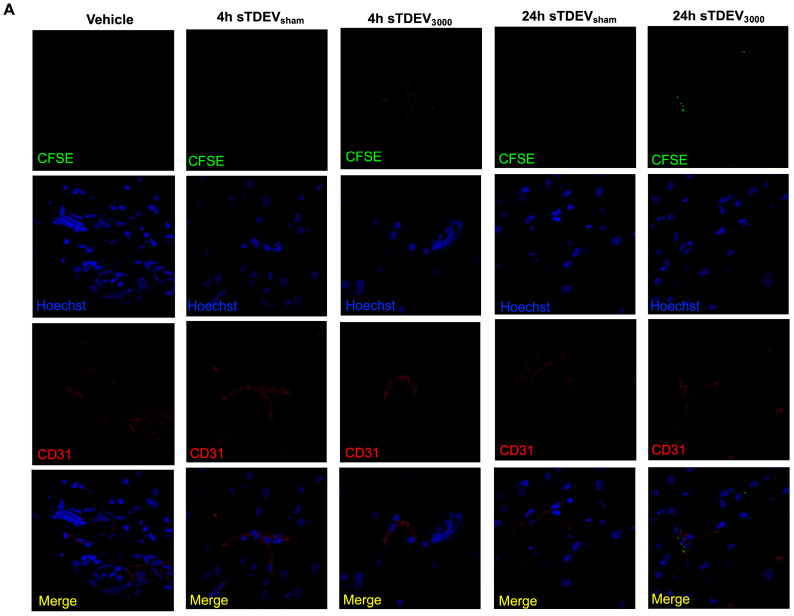
sTDEVs released after ablative H-FIRE doses accumulate near cerebral endothelial cells in vivo. sTDEVs were isolated from immediately post-H-FIRE supernatants of 0 V/cm- or 3000 V/cm-treated F98 glioma cells. sTDEVs were labeled with CFSE and administered via intracranial infusion to healthy Fischer rats. Endpoints were 4 and 24 h after infusion. (**A**) Representative confocal microscopy images of brain tissue showing exosomal distribution relative to CD31^+^ cells (100× magnification). sTDEVs are visible in green, endothelial cells are visible in red, and nuclei are visible in blue.

In vitro, disruptive sTDEVs were selectively internalized compared to non-disruptive sTDEVs by cerebral endothelial cells. We therefore sought to determine whether disruptive sTDEVs exhibited this same cerebral endothelial cell tropism compared to non-disruptive sTDEVs in vivo. Confocal microscopy demonstrated that non-disruptive sTDEVs, which were scarcely visible in the brain both 4 and 24 h post-infusion, did not accumulate in or near cerebral endothelial cells (Figure 6). Disruptive sTDEVs, released by 3000 V/cm H-FIRE-treated glioma cells, accumulated near cerebral endothelial cells and endothelium-lined structures both 4 and 24 h after infusion (Figure 6). Taken together with the preferential uptake of disruptive sTDEVs by cerebral endothelial cells in vitro and the significantly altered proteomic payloads of disruptive sTDEVs, these data suggest that H-FIRE ablation of glioma cells alters proteomic profiles of sTDEVs, resulting in altered brain- and cerebral endothelial cell tropism in vivo.

### 3.5. sTDEVs Released by H-FIRE-Treated Glioma Cells Promote Infiltration of Iba1+ Cells

**Figure 7:**
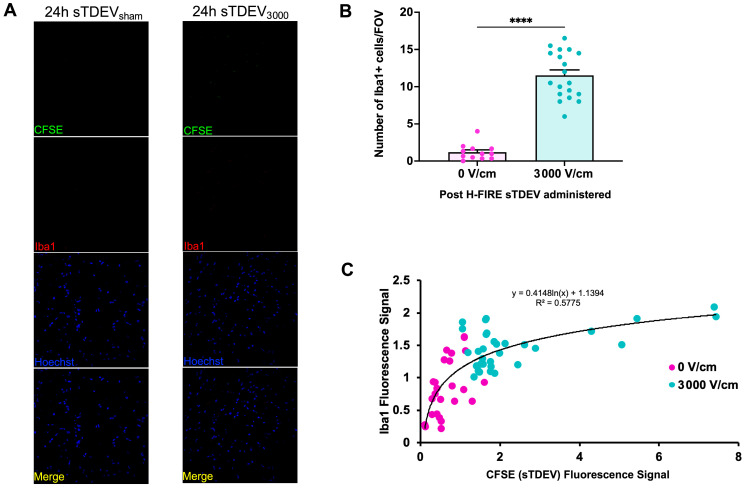
Presence of sTDEVs released by H-FIRE-treated glioma cells correlates with infiltration of Iba1+ cells in the brain. Animals received intracranial injections of CFSE-labeled sTDEVs released by 0 V/cm- and H-FIRE-treated glioma cells (n = 3 and n = 2, respectively). Confocal microscopy of tissues harvested 24 h post-treatment was used to assess the presence of Iba1+ cells in the brain compared to the presence of CFSE-labeled sTDEVs. (**A**) Representative confocal microscopy images of brain tissue showing CFSE-labeled sTDEVs (green) and Iba1+ cells (red) (100× magnification). (**B**) Quantification of the number of Iba1+ cells per field of view in animals that received sTDEVs released by 0 V/cm-treated glioma cells vs. sTDEVs released by 3000 V/cm H-FIRE-treated glioma cells. Statistical analysis using an unpaired t-test. **** *p* ≤ 0.0001. n = 3 received 0 V/cm sTDEVs, and n = 2 received 3000 V/cm sTDEVs. (**C**) Logarithmic fit of CFSE (sTDEV) fluorescence signal plotted against Iba1 fluorescence signal.

Because increased cerebrotropism and endothelial cell tropism were observed in sTDEVs released by H-FIRE-treated glioma cells, we hypothesized that these sTDEVs may contribute to the recruitment of immune scavenger cells from the vasculature into the brain. We quantified the number of Iba1+ cells per field of view in animals that received sTDEVs released by 0 V/cm- and H-FIRE-treated glioma cells (n = 3 and n = 2, respectively) 24 h post-administration to determine whether sTDEVs released after H-FIRE ablation of glioma, which are maintained in the brain, may contribute to the local immune response via macrophage recruitment. Confocal microscopy revealed that animals receiving disruptive sTDEVs, those released from 3000 V/cm H-FIRE-treated glioma cells, had significantly increased numbers of Iba1+ cells per field of view in the brain compared to animals that received non-disruptive sTDEVs released by 0 V/cm-treated glioma cells (Figure 7A,B). In order to determine whether the increase in Iba1+ cells is driven by the presence of post-H-FIRE sTDEVs in the brain, we performed a logarithmic fit to assess whether increased sTDEVs (CFSE fluorescence intensity signal) correlated with increased Iba1 fluorescence intensity signal. The logarithmic fit yielded an R^2^ value of 0.5775, with a clear distinction between sTDEVs released by 0 V/cm-treated glioma cells and those released by H-FIRE-treated glioma cells (Figure 7C). The highest CFSE fluorescence signals and highest Iba1+ fluorescence signals were recorded in animals that received sTDEVs released by H-FIRE-treated glioma cells, while the lowest CFSE fluorescence signals and lowest Iba1+ fluorescence signals were recorded in animals that received sTDEVs released by 0 V/cm-treated glioma cells (Figure 7C). Together, the data suggest that retention of sTDEVs released by H-FIRE-treated glioma cells in the brain correlates with an increased presence of Iba1+ cells in the brain. This suggests that sTDEVs released after H-FIRE ablation of glioma are retained in the brain and may contribute to the local microglia cell recruitment.

### 3.6. sTDEVs Released by H-FIRE-Treated Glioma Cells Do Not Disrupt the BBB In Vivo

**Figure 8:**
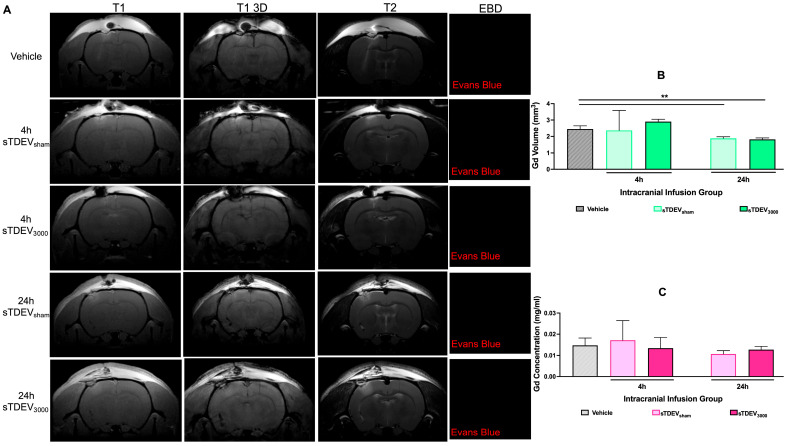
sTDEVs released after ablative H-FIRE doses do not disrupt the blood–brain barrier in vivo. sTDEVs were isolated from immediately post-H-FIRE supernatants of 0 V/cm- or 3000 V/cm-treated F98 glioma cells. sTDEVs were administered via intracranial infusion to healthy Fischer rats. Prior to the endpoint (4 and 24 h post-infusion), animals received Evans Blue Dye and gadolinium prior to MRI and euthanasia. (**A**) Representative T1-, T1 3D, and T2-weighted MRI images and confocal microscopy images of Evans Blue Dye distribution in the plane of the intracranial infusion. (**B**) Quantification of gadolinium-enhancing volume. (**C**) Quantification of gadolinium concentration in brain tissue. One-way ANOVA with Tukey’s post hoc test was used for statistical analysis. ** *p* ≤ 0.01. n ≥ 3 for all groups.

Previous work demonstrates that sTDEVs released by 3000 V/cm H-FIRE-treated glioma cells disrupt the BBB endothelium in vitro. To determine whether these in vitro-disruptive sTDEVs disrupt the in vivo BBB, animals received Evans Blue Dye and gadolinium contrast enhancement and underwent T1, 3D T1, and T2 MRI 4 and 24 h post-infusion. Contrast enhancement was visible along the infusion insertion tract in all animals and was localized to the region of the infusion (Figure 8A). Regions of contrast enhancement visible on T1 and 3D T1 scans spatially coincided with hyperintense regions on T2 sequences (Figure 8A), suggesting that the observed BBB disruption is primarily attributable to inflammation caused by the infusion. Evans Blue Dye was not visible in confocal microscopy images of the infusion zone (Figure 8A). Quantification of gadolinium-enhancing volume in T1 sequences demonstrates that there was no significant difference in gadolinium-enhancing volumes between animals receiving saline vehicle injection, sTDEVs released by 0 V/cm-treated glioma cells, and sTDEVs released from 3000 V/cm-treated glioma cells at the 4 h timepoint. There was no significant difference in gadolinium-enhancing volume between animals receiving sTDEVs released by 0 V/cm-treated glioma cells and those receiving sTDEVs released from 3000 V/cm-treated glioma cells at the 24 h timepoint. There was a significant decrease in gadolinium-enhancing volume in the vehicle and 0 V/cm sTDEV animals at the 24 h timepoint compared to the vehicle animals (*p* ≤ 0.01) (Figure 8B). This suggests that infusions induced gadolinium enhancement at early timepoints that then recovered by 24 h post-infusion. Quantification of gadolinium concentration in the brain revealed no significant differences in gadolinium concentration between any of the treatment groups (Figure 8C). Together, these results suggest that the in vitro–BBB disruptive sTDEVs released by 3000 V/cm H-FIRE-treated glioma cells do not disrupt the BBB in vivo.

## 4. Discussion

Following brain tumor ablation with H-FIRE, the pBBB is focally and reversibly disrupted. The mechanisms underlying this post-ablation pBBB disruption are incompletely uncharacterized. A complete understanding of these mechanisms will inform the mechanism-driven design of multimodal treatment strategies, employing H-FIRE ablation of the tumor bulk and rational delivery of systemic therapeutics to treat infiltrative tumor margins. Extracellular vesicles represent a major mechanism of communication within the tumor microenvironment, and it is known that anticancer therapies can alter tumor-derived extracellular vesicles to alter the tumor microenvironment. We have previously demonstrated that after treatment with an ablative 3000 V/cm dose of H-FIRE, primary and metastatic brain cancer cells (F98 glioma and LL/2 Lewis lung carcinoma cells, respectively) immediately release small tumor-derived extracellular vesicles that increase permeability of the BBB endothelium in vitro (“disruptive” sTDEVs) compared to sTDEVs released after 0 and 1500 V/cm H-FIRE treatment (“non-disruptive” sTDEVs) [14]. This mechanism was unique to H-FIRE, as sTDEVs released by irradiated tumor cells did not alter the permeability of the BBB endothelium model.

Here, our results indicate that the sTDEV populations released after H-FIRE treatment of F98 and LL/2 cancer cells contain sTDEVs, a specific subtype of extracellular vehicle, and have significantly altered proteomic payloads. Mass spectrometry proteomic analysis of sTDEVs released after H-FIRE treatment of F98 glioma cells demonstrates that the disruptive sTDEVs, released after the ablative 3000 V/cm H-FIRE dose, have distinct proteomic profiles compared to the non-disruptive sTDEVs released after lower doses of H-FIRE and 0 V/cm treatment. Specifically, 108 proteins are unique to the disruptive sTDEVs and are not present in the “non-disruptive” sTDEVs, indicating that H-FIRE significantly alters proteomic payloads of sTDEVs released after tumor cell ablation. To determine a possible mechanism of the BBB endothelium disruption observed previously, we used confocal microscopy to measure uptake of fluorescently labeled post-H-FIRE sTDEVs by cerebral endothelial cell monolayers. These results demonstrate that only sTDEVs released by tumor cells treated with the ablative 3000 V/cm dose of H-FIRE, or the “disruptive” sTDEVs from our previous in vitro investigations, were internalized by cerebral endothelial cells in vitro. In vivo, when delivered via intracranial infusion, the disruptive sTDEVs are retained in the brain. This is in contrast to non-disruptive sTDEVs released after 0 V/cm H-FIRE treatment of glioma cells, which are scarcely detectable in the brain both 4 and 24 h after treatment, despite their visible accumulation in the spleen and liver. Similarly to in vitro, the disruptive sTDEVs exhibit increased cerebral endothelial cell tropism compared to non-disruptive sTDEVs. We also demonstrate that animals that received disruptive sTDEVs had an increased presence of Iba1+ cells in the brain compared to animals that received non-disruptive sTDEVs. Further, the presence of administered sTDEVs in the brain correlates with increased presence of Iba1+ cells. Finally, our results demonstrate that the in vitro-disruptive sTDEVs do not disrupt the BBB in vivo.

Taken together, we have demonstrated that after H-FIRE ablation of glioma, cells release populations of extracellular vesicles containing sTDEVs, which have significantly altered proteomic payloads, increased brain- and endothelial cell tropism, and correlate with increased Iba1+ cell infiltration compared to sTDEVs released by 0 V/cm-treated glioma cells. To the best of the authors’ knowledge, this is the first study demonstrating that electroporation-based ablation therapies, such as H-FIRE, functionally and proteomically alter tumor-derived extracellular vesicles. Previous investigations of the effects of tumor ablation therapies on tumor-derived extracellular vesicle composition and function are limited. Sheybani et al. demonstrated that thermal focused ultrasound ablation of glioma cells significantly increases the release of sTDEVs and alters the exosomal proteomic payloads and that sTDEVs released after focused ultrasound ablation increased dendritic cell production of IL-12p70 [19]. Investigations of other thermal ablation modalities, including general heat stress, cryoablation, and photodynamic therapy, indicate that thermal ablation of tumor cells may alter sTDEVs to induce bystander effects in neighboring cells [20,21,22]. Fewer studies have investigated the effects of nonthermal modalities, such as electroporation- and pulsed electric field-based therapies, on tumor-derived extracellular vesicles. Microbubble-assisted ultrasound treatment of head and neck cancer cells has been shown to increase the release of extracellular vesicles [23]. Fukuta demonstrated that low-level, steady-state electrical stimulation (0.34 mA/cm^2^) of murine melanoma and fibroblast cells increased the secretion of extracellular vesicles in both cell lines without any changes in uptake of these vesicles by their donor cells [24]. Prevc et al. demonstrated that microvesicles released by electroporated (800 V/cm) canine melanoma cells may trigger bystander cell death in naïve melanoma cells [25]. Other types of cellular and microenvironmental stress, such as hypoxia, have been shown to alter tumor-derived extracellular vesicles. Zhao et al. demonstrated that sTDEVs released from hypoxic glioma cells increased BBB permeability in mice compared to those released from normoxic glioma cells [13]. Here, our results suggest that in addition to the direct ablative effects of H-FIRE, tumor-derived extracellular vesicles released by treated tumor cells are proteomically and tropically altered to function as vehicles of communication within the post-ablation tumor microenvironment.

Specifically, the presence of exosomal signatures in the post-H-FIRE sTDEV populations suggests that these sTDEV populations contain exosomes; however, further characterization is necessary to identify potential subpopulations with functional significance. Our study is also limited to exclusively characterizing exosomal markers, and future work should include marker characterization for other extracellular vesicle subtypes, including microvesicles. Additionally, the significantly altered proteomic profiles of the disruptive sTDEVs, released after treatment of glioma cells with 3000 V/cm H-FIRE, indicate that H-FIRE significantly alters proteomic cargo packaging within tumor-derived extracellular vesicles within the first 20 min of H-FIRE treatment. This is in agreement with investigations by Sheybani et al., demonstrating that focused ultrasound thermal ablation of glioma cells alters sTDEV proteomic profiles within the first 15 min of treatment [19]. Ionizing radiation has been shown to alter the proteomic cargo of head and neck cancer cells within the first 18–24 h of treatment [26,27,28]. Pathways most highly associated with the 108 proteins unique to the disruptive sTDEVs include various integrin signaling pathways, PDGFR signaling, and ubiquitination and SUMOylation processes. Because these sTDEVs disrupted the BBB endothelium in vitro, such pathways represent potential mechanisms of post-H-FIRE sTDEV-mediated functional modification of cerebral endothelial cells. Partridge et al. previously demonstrated increased ubiquitination of key BBB proteins when H-FIRE was applied to healthy brain tissue [29]. Here, we demonstrate increased ubiquitin- and SUMOylation-associated proteins in sTDEVs released by H-FIRE-treated glioma cells. Ubiquitination and SUMOylation are well-documented post-translational modifications marking proteins for degradation. Taken together with Partridge et al.’s results in healthy brain tissue, it is possible that H-FIRE may alter extracellular vesicle cargo in a cancer-independent manner, whereby H-FIRE-treated cancerous or noncancerous cells release extracellular vesicles that mediate ubiquitination and SUMOylation of key BBB proteins. Future work will characterize ubiquitination of cerebral endothelial cells exposed to sTDEVs released by H-FIRE-treated glioma cells and proteomic profiles of extracellular vesicles released by non-cancerous cells treated with H-FIRE. Future work will also characterize proteomic payloads of sTDEVs at later timepoints post-H-FIRE to determine whether sTDEVs may mediate microenvironmental changes long after ablation.

Although our data demonstrate that H-FIRE proteomically alters sTDEVs, and these sTDEVs exhibit increased retention in the brain and cerebral endothelial cell tropism, our study is limited in the administration of these post-H-FIRE sTDEVs to healthy brains. Investigations into the role of these post-H-FIRE sTDEVs in tumor-bearing brains are warranted. Further, our in vitro cerebral endothelial cell uptake results and in vivo brain and endothelial cell tropism results suggest that sTDEVs released after H-FIRE treatment of tumor cells contain surface modifications that mediate these tropic effects. However, future work should investigate the tropism of these sTDEVs with respect to other brain cell types, including astrocytes and microglia. Lysosomal markers may also be employed in future investigations to confirm internalization of sTDEVs by endothelial cells. Our results demonstrate global proteomic changes and specific proteins unique to the sTDEVs released after H-FIRE treatment of parent tumor cells, but future investigations are needed to determine which of these proteins are present on the exosomal surface and may mediate these tropic effects. Because this work does not directly and functionally validate any specific sTDEV proteins responsible for the observed effects, the use of recombinant vesicles or neutralizing assays may be used to identify functionally significant proteomic cargos in the sTDEVs released by H-FIRE-ablated tumor cells. Future work may also utilize orthotopic primary tumor models to investigate H-FIRE-induced sTDEV proteomic changes following in vivo tumor ablation and may seek to compare proteomic differences between F98- and LL/2-derived post-H-FIRE sTDEVs.

The fact that sTDEVs released after H-FIRE treatment of parent tumor cells are retained in the brain more significantly than sTDEVs released by 0 V/cm-treated glioma cells suggests that these sTDEVs may mediate microenvironmental changes within the brain after brain tumor ablation via H-FIRE. Here, we demonstrate that animals that received the sTDEVs released by H-FIRE-treated glioma cells, which are more significantly retained in the brain compared to sTDEVs released by 0 V/cm-treated cells, have an increased presence of Iba1+ cells in the brain. This suggests that the release of proteomically altered sTDEVs after H-FIRE ablation of glioma may be a mechanism by which macrophages are recruited to the post-ablation site to mediate a local immune response. However, additional work is needed in order to determine whether these are systemically recruited macrophages or local microglia. There is a logarithmic, positive correlation between sTDEV fluorescence signal and Iba1 fluorescence signal. Notably, the highest sTDEV signals and the highest Iba1 signals are observed in animals that received disruptive sTDEVs, while the lowest sTDEV and Iba1 signals are observed in animals that received non-disruptive sTDEVs. Confocal imaging in this study served as a qualitative assessment of tissue-level fluorescence following administration of sTDEVs from H-FIRE–treated glioma cells. The data were not intended to resolve cellular morphology or subcellular localization but to demonstrate that pulsed electric field exposure can influence EV composition and in vivo behavior. While the absence of morphological detail limits interpretation, and fluorescence-based measures may include non-specific signals, all groups were imaged under identical acquisition parameters, permitting valid relative comparisons. These findings therefore provide foundational evidence that H-FIRE modulates EV cargo and bioactivity, supporting further mechanistic studies using multiplexed and high-resolution imaging approaches. Together, these data suggest that the increase in Iba1+ cells may be driven by the administered sTDEVs and that sTDEVs released by H-FIRE-treated glioma cells are functionally unique in promoting this infiltration compared to sTDEVs released by 0 V/cm-treated glioma cells. Future work may include more comprehensive immune cell staining to determine the change in immune infiltration. Future investigations may also include ex vivo studies to determine the impact of H-FIRE-induced sTDEVs on immune cell migration in various immune cell lines.

Although our results show that these sTDEVs do not increase in vivo permeability of the BBB under our experimental conditions, these post-H-FIRE sTDEVs are retained in the brain, exhibit cerebral endothelial cell tropism, and correlate with increased infiltration of Iba1+ cells; thus, future investigations are warranted to determine the timescale of this retention and their furthered roles in the brain microenvironment after H-FIRE ablation of brain tumors. Future investigations will identify the timescale of sTDEV-mediated Iba1+ cell recruitment into the brain and will investigate polarization of recruited macrophages. Our results also demonstrate that, although post-H-FIRE sTDEVs are retained in the brain more significantly than sTDEVs released by 0 V/cm-treated glioma cells, the post-H-FIRE sTDEVs are detectable in other organs, including the liver and spleen. Future work may seek to utilize engineered tumor cell lines releasing fluorescently tagged sTDEVs to characterize the biodistribution of sTDEVs released by in vivo H-FIRE-treated brain tumors.

Pulsed electric field-mediated cytoskeletal remodeling and tight junction reorganization are established contributors to H-FIRE-induced BBB disruption, particularly within lower-field penumbra regions. Our results extend this model by identifying a second, field-conditioned mechanism: ablative H-FIRE rapidly alters the proteome and tropism of sTDEVs, which are subsequently retained in the brain and engage with the endothelium and Iba1+ cells without independently increasing in vivo BBB permeability. We therefore posit a two-phase mechanism in which H-FIRE provides an immediate, field strength-dependent opening of the BBB, while H-FIRE-conditioned sTDEVs modulate the post-ablation microenvironment cellular responses. Future studies, including field-only, sTDEV-only, and field + sTDEVs, and pathway-specific blockades (integrins, PDGFR, ubiquitin), are needed to test this potential synergy.

A complete understanding of microenvironmental modifications after H-FIRE ablation of brain tumors is critical for its continued translation and rational design of multimodal treatment approaches combining H-FIRE tumor ablation and adjuvant therapeutics. Extracellular vesicles are a known mechanism by which cancer cells communicate with their microenvironment, and it is known that antineoplastic therapies can alter tumor-derived extracellular vesicles to functionally modify the tumor microenvironment. These data demonstrate that H-FIRE, a novel nonthermal tumor ablation therapeutic, alters the proteomic payloads of extracellular vesicles, which then exhibit increased retention in the brain, endothelial cell tropism, and correlation with increased infiltration of Iba1+ cells. This data furthers our mechanistic understanding of tumor microenvironment modulation post-H-FIRE, whereby H-FIRE modifies sTDEVs to be retained in the brain and facilitate further modulation of the brain tumor microenvironment after ablation of the tumor bulk.

## Figures and Tables

**Table 1:** Signaling pathways most related to the 108 proteins unique to the “disruptive” sTDEVs. Proteins were submitted for pathway analysis in Enrichr using the NCI Nature 2016, Bioplanet 2019, Elsevier Pathway Collection, and Panther 2016 Libraries.

NCI Nature 2016	Bioplanet 2019	Elsevier Pathway Collection	Panther 2016
Regulation of CDC42 activity	Proteins involved in Angelman Syndrome	Antigen presentation: folding, assembly, and peptide loading of class I MHC proteins	PDGF signaling pathway
Alpha4 beta1 integrin signaling events	PDGFR->FOXO3A signaling	Ubiquitin-mediated proteolysis	Insulin/IGF pathway-protein kinase B signaling cascade
Signaling events mediated by TCPTP	Advanced glycation end products in diabetes or age-related erectile dysfunction	COPII-mediated vesicle transport	Ubiquitin proteasome pathway
Beta3 integrin cell surface interactions	MTOR signaling activation by fatty acids and glucose	PIP biosynthesis at the late endosome membrane	p53 pathway feedback loops 2
PDGF receptor signaling network	Golgi transport	Deadenylation-dependent mRNA decay	p53 pathway feedback loops 2
A4b7 integrin signaling	Ca^2+^ overload in muscular dystrophies		Integrin signaling pathway
TCR signaling in the naive CD4^+^ TCR pathway	MRNA degradation		Ras pathway
	Vascular endothelial cell vasodilation in the brain		p53 pathway
	Dystrophin glycoprotein complex signaling in muscular dystrophies		DNA replication
	Vascular mobility		FGF signaling pathway

## Data Availability

The original contributions presented in this study are included in the article/Supplementary Materials. Further inquiries can be directed to the corresponding author(s).

## Supplementary Materials

The following supporting information can be downloaded at: https://www.mdpi.com/article/10.3390/cells14221782/s1, Figure S1: STDEV were isolated from immediately post-treatment supernatants of F98 glioma cells treated with 0, 1500, and 3000 V/cm H-FIRE and characterized via label-free, bottom-up LC-MS/MS proteomics; Figure S2: sTDEV were isolated from immediately post-H-FIRE supernatants of sham- or 3000 V/cm-treated F98 glioma cells.

## Abbreviations

The following abbreviations are used in this manuscript:

H-FIRE	High-frequency irreversible electroporation
BBB	Blood–brain barrier
sTDEV	Small tumor-derived extracellular vesicle
BM	Brain metastasis
GBM	Glioblastoma multiforme
IRE	Irreversible electroporation
DMEM	Dulbecco’s modified eagle medium
CFSE	Carboxyfluorescein succinimidyl ester
EBD	Evans Blue Dye
CEV	Contrast-enhancing lesion volumes
MRI	Magnetic resonance imaging
PBS	Phosphate-buffered saline
