# Supplementary material for: High-Frequency Irreversible Electroporation Alters Proteomic Profiles and Tropism of Small Tumor-Derived Extracellular Vesicles to Promote Immune Cell Infiltration
Source: Cells. 2025 Nov 13;14(22):1782. doi: 10.3390/cells14221782 (PMC12651280; doi:10.3390/cells14221782)
Supplement: Supplementary file 1 [file cells-14-01782-s001.zip › cells-3950443-supplementary.pdf]

Symbol	Protein Name	Symbol	Protein Name	Symbol	Protein Name	Symbol	Protein Name
AARSD1	alanyl-tRNA synthetase domain containing 1	GAA	alpha glucosidase	ACP1	NADH:ubiquinone oxidoreductase subunit AB1	SNRNP27	small nuclear ribonucleoprotein U4/U6.U5 subunit 27
ABCF3	ATP binding cassette subfamily F member 3	GET4	guided entry of tail-anchored proteins factor 4	NKAPD1	NKAP domain containing 1	SPAST	spastin
ADPRHL2	ADP-ribosylserine hydrolase	GIT1	GIT ArfGAP 1	SF1	nuclear receptor subfamily 5 group A member 1	SPPL2B	signal peptide peptidase like 2B
AGAP1	ArfGAP with GTPase domain, ankyrin repeat and PH domain 1	GMPR2	guanosine monophosphate reductase 2	NUFIP2	nuclear FMR1 interacting protein 2	SREK1IP1	SREK1 interacting protein 1
AGAP3	ArfGAP with GTPase domain, ankyrin repeat and PH domain 3	GPX4	glutathione peroxidase 4	OLFML3	olfactomedin like 3	SRRT	serrate, RNA effector molecule
AKAP13	A-kinase anchoring protein 13	GSTA4	glutathione S-transferase alpha 4	PDGFB	platelet derived growth factor subunit B	TATDN1	TatD DNase domain containing 1
ANGPTL2	angiopoietin like 2	GTF2F1	general transcription factor IIF subunit 1	PDPK1	3-phosphoinositide dependent protein kinase 1	TBC1D10A	TBC1 domain family member 10A
ARL2	ADP ribosylation factor like GTPase 2	HAPLN1	hyaluronan and proteoglycan link protein 1	PDS5A	PDS5 cohesin associated factor A	TINAGL1	tubulointerstitial nephritis antigen like 1
ATOX1	antioxidant 1 copper chaperone	HERC2	HECT and RLD domain containing E3 ubiquitin protein ligase 2	PGPEP1	pyroglutamyl-peptidase I	TMBIM1	transmembrane BAX inhibitor motif containing 1
CALD1	caldesmon 1	HTRA1	HtrA serine peptidase 1	PHF5A	PHD finger protein 5A	TMEM87A	transmembrane protein 87A
CDC16	cell division cycle 16	HUWE1	HECT, UBA and WWE domain containing E3 ubiquitin protein ligase 1	PIH1D1	PIH1 domain containing 1	TNPO3	transportin 3
CDC73	cell division cycle 73	IFT122	intraflagellar transport 122	PIK3C3	phosphatidylinositol 3-kinase catalytic subunit type 3	TNS1	tensin 1
CLIP2	CAP-Gly domain containing linker protein 2	INTS3	integrator complex subunit 3	PLAA	phospholipase A2 activating protein	TRIP12	thyroid hormone receptor interactor 12
CNOT9	CCR4-NOT transcription complex subunit 9	IQCE	IQ motif containing E	POLR2D	RNA polymerase II subunit D	TTC21B	tetratricopeptide repeat domain 21B
CPPED1	calcineurin like phosphoesterase domain containing 1	ITGA4	integrin subunit alpha 4	POLR3A	RNA polymerase III subunit A	TUBGCP4	tubulin gamma complex associated protein 4
DAD1	defender against cell death 1	KDELR1	KDEL endoplasmic reticulum protein retention receptor 1	PRIM1	DNA primase subunit 1	TUBGCP6	tubulin gamma complex associated protein 6
DBNL	drebrin like	LAMC1	laminin subunit gamma 1	PRPF3	pre-mRNA processing factor 3	UBE2A	ubiquitin conjugating enzyme E2 A
DGKA	diacylglycerol kinase alpha	LARP4B	La ribonucleoprotein 4B	PUS7	pseudouridine synthase 7	UBE3A	ubiquitin protein ligase E3A
DOCK6	dedicator of cytokinesis 6	LRCH3	leucine rich repeats and calponin homology domain containing 3	RASA2	RAS p21 protein activator 2	URI1	URI1 prefoldin like chaperone
EFCAB7	EF-hand calcium binding domain 7	LSM1	LSM1 homolog, mRNA degradation associated	RBM26	RNA binding motif protein 26	USP16	ubiquitin specific peptidase 21
EIF2AK2	eukaryotic translation initiation factor 2 alpha kinase 2	LSM12	LSM12 homolog	RBM42	RNA binding motif protein 42	USP8	ubiquitin specific peptidase 8
ELP4	elongator acetyltransferase complex subunit 4	MAP7D1	MAP7 domain containing 1	RPAP3	RNA polymerase II associated protein 3	UTRN	utrophin
EML4	EMAP like 4	MBNL2	muscleblind like splicing regulator 2	RRP1B	ribosomal RNA processing 1B	VCAN	versican
EVA1B	eva-1 homolog B	MCRIP1	MAPK regulated corepressor interacting protein 1	SCARB1	scavenger receptor class B member 1	XRN1	5'-3' exoribonuclease 1
EXOSC2	exosome component 2	MKI67	marker of proliferation Ki-67	SEC13	SEC13 homolog, nuclear pore and COPII coat complex component	YPEL5	yippee like 5
FCSK	fucose kinase	MTHFD1L	methylenetetrahydrofolate dehydrogenase (NADP+ dependent) 1 like	SEC24C	SEC24 homolog C, COPII coat complex component	ZRANB2	zinc finger RANBP2-type containing 2
FIG4	FIG4 phosphoinositide 5-phosphatase	NAA30	N-alpha-acetyltransferase 30, NatC catalytic subunit	SMG1	SMG1 nonsense mediated mRNA decay associated PI3K related kinase		

Supplemental Figure S1. STDEV were isolated from immediately post-treatment supernatants of F98 glioma cells treated with 0, 1,500, and 3,000 V/cm H-FIRE and characterized via label-free, bottom-up LC-MS/MS proteomics. “Non-disruptive” grouping includes tumor-derived sTDEV released after 0 and 1,500 V/cm H-FIRE treatment which did not disrupt a Transwell® model of the BBB endothelium,[14] while “disruptive” grouping includes tumor-derived sTDEV released after 3,000 V/cm H-FIRE treatment which increased permeability of this model. List of the 108 proteins unique to glioma-derived sTDEV released after the 3,000 V/cm H-FIRE dose (“disruptive” sTDEV) were submitted to Enrichr for pathway analysis

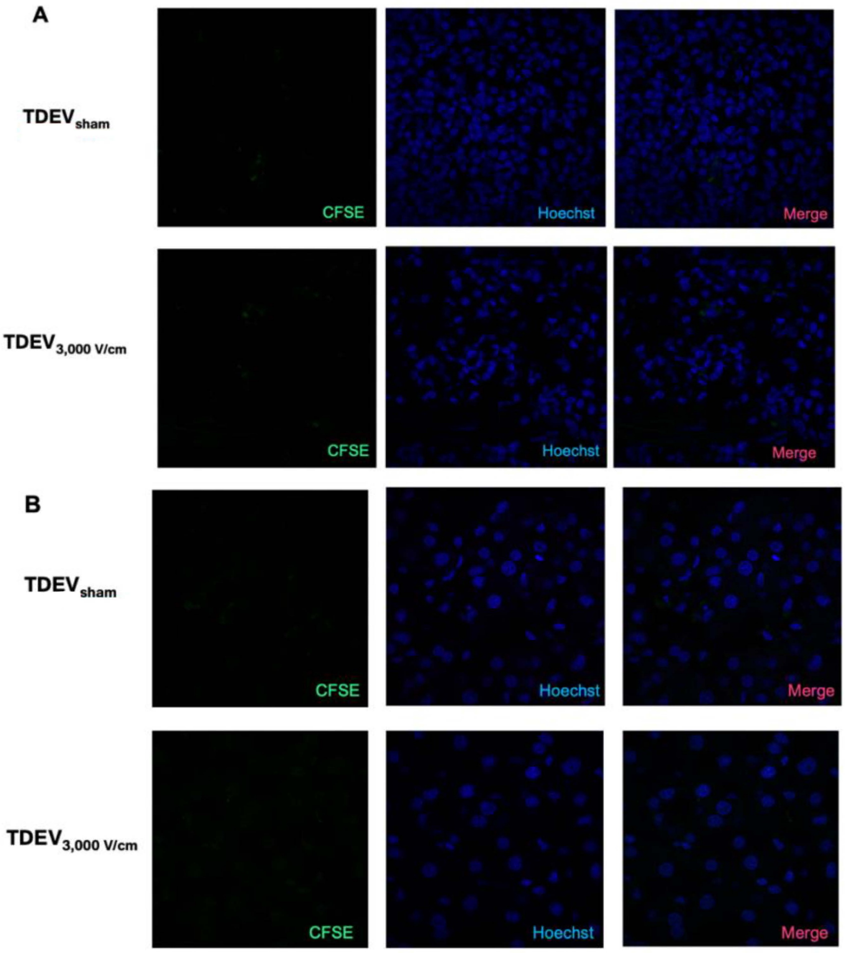

Supplemental Figure S2. sTDEV were isolated from immediately post-H-FIRE supernatants of sham- or 3,000 V/cm-treated F98 glioma cells. sTDEV were labelled with CFSE, and administered via intracranial infusion to healthy Fischer rats. Endpoints were 4 and 24 hours after infusion. Representative confocal microscopy images of brain tissue showing exosomal distribution in the spleen **(a)** and liver **(b).** sTDEV are visible in green and nuclei are visible in blue.
